# Diagnosis of ADHD using virtual reality and artificial intelligence: an exploratory study of clinical applications

**DOI:** 10.3389/fpsyt.2024.1383547

**Published:** 2024-06-03

**Authors:** Soohwan Oh, Yoo-Sook Joung, Tai-Myoung Chung, Junho Lee, Bum Joon Seok, Namuk Kim, Ha Min Son

**Affiliations:** ^1^ Department of Psychiatry, Samsung Changwon Hospital, Sungkyunkwan University School of Medicine, Changwon, Republic of Korea; ^2^ Department of Psychiatry, Samsung Medical Center, Sungkyunkwan University School of Medicine, Seoul, Republic of Korea; ^3^ Hippo T&C, Suwon, Republic of Korea; ^4^ Department of Computer Science and Engineering, College of Computing, Sungkyunkwan University, Suwon, Republic of Korea; ^5^ Department of Computer Science, University of California, Davis, Davis, United States

**Keywords:** attention deficit hyperactivity disorder (ADHD), virtual reality (VR), artificial intelligence (AI), diagnosis of ADHD, digital therapeutics, digital diagnostic device

## Abstract

**Introduction:**

Diagnosis of Attention Deficit/Hyperactivity Disorder (ADHD) is based on clinical evaluation of symptoms by a psychiatrist, referencing results of psychological tests. When diagnosing ADHD, the child’s behavior and functionality in real-life situations are critical components. However, direct observation by a clinician is often not feasible in practice. Therefore, such information is typically gathered from primary caregivers or teachers, which can introduce subjective elements. To overcome these limitations, we developed AttnKare-D, an innovative digital diagnostic tool that could analyze children’s behavioral data in Virtual Reality using Artificial Intelligence. The purpose of this study was to explore the utility and safety of AttnKare-D for clinical application.

**Method:**

A total of 21 children aged between 6 and 12 years were recruited for this study. Among them, 15 were children diagnosed with ADHD, 5 were part of a normal control group, and 1 child was excluded due to withdrawal of consent. Psychological assessments, including K-WISC, Conners CPT, K-ARS, and K-CBCL, were conducted for participants and their primary caregivers. Diagnoses of ADHD were confirmed by child and adolescent psychiatrists based on comprehensive face-to-face evaluations and results of psychological assessments. Participants underwent VR diagnostic assessment by performing various cognitive and behavioral tasks in a VR environment. Collected data were analyzed using an AI model to assess ADHD diagnosis and the severity of symptoms.

**Results:**

AttnKare-D demonstrated diagnostic performance with an AUC of 0.893 when compared to diagnoses made by child and adolescent psychiatrist, showing a sensitivity of 0.8 and a specificity of 1.0 at a cut-off score of 18.44. AttnKare-D scores showed a high correlation with K-ARS scores rated by parents and experts, although the correlation was relatively low for inattention scores.

**Conclusion:**

Results of this study suggest that AttnKare-D can be a useful tool for diagnosing ADHD in children. This approach has potential to overcome limitations of current diagnostic methods, enhancing the accuracy and objectivity of ADHD diagnoses. This study lays the groundwork for further improvement and research on diagnostic tools integrating VR and AI technologies. For future clinical applications, it is necessary to conduct clinical trials involving a sufficient number of participants to ensure reliable use.

## 1 Introduction

Attention Deficit/Hyperactivity Disorder (ADHD), a neurodevelopmental disorder characterized by inattention, hyperactivity, and impulsivity, typically manifests before the age of 12. It often leads to chronic progress and functional impairments in various areas such as family, school, and social contexts (1). The prevalence of ADHD varies by country and period. It is relatively high worldwide, affecting about 5.29% of the general population (2). Early diagnosis and continuous treatment of ADHD are crucial as they can effectively control symptoms and minimize functional decline (3).

Clinicians diagnose ADHD in a clinical setting based on interviews with the patient and parents, observation of the patient, and reports from parents and teachers using their professional knowledge (4). To obtain more objective data for diagnosis, various assessment tools can be used. Common tools for evaluating ADHD symptoms include the ADHD Rating Scale, 4th edition (ARS-IV), and the Revised Conners’ Parent Rating Scale (CPRS-R), which are also useful for assessing symptom severity (5, 6). Neuropsychological tests to assess attention, concentration, impulsivity, and cognitive flexibility include the Continuous Performance Test (CPT), Stroop test, and Trail Making Test (7, 8). Additionally, intelligence tests and Child Behavior Check List (CBCL) can be used to assess psychiatric symptoms that may be influenced by or indicative of ADHD symptoms (9).

Despite the availability of various assessment aids, the diagnostic process for ADHD demands substantial expertise for several reasons. Firstly, the optimal method for forming a diagnostic impression—direct observation of a child’s behavior and activities outside the clinical environment—is often impractical. As a result, clinicians predominantly rely on second-hand accounts from parents or teachers. Such indirect information, potentially subjective in nature, requires clinicians to analyze it carefully and thoughtfully. Secondly, the majority of neuropsychological assessments conducted in controlled clinical settings may not accurately reflect a child’s natural behavior or symptom manifestation in daily life. Due to limited efficacy of these tests alone, it is crucial for clinicians to incorporate observed clinical symptoms into their diagnosis, ensuring a more comprehensive and accurate assessment of ADHD (10–12). Lastly, core symptoms of ADHD, such as inattention, hyperactivity, and impulsivity, are known to manifest in a significant proportion of the pediatric population (20-57%) and subject to influence by other psychiatric conditions. Consequently, a nuanced understanding of a child’s cognitive and emotional development, alongside consideration of co-occurring psychiatric disorders, is crucial in the ADHD diagnostic process (13).

Advancements in digital technology have enabled the utilization of Virtual Reality (VR) to construct specific environments for objective assessment of human behaviors. This technology transcends limitations of physical spaces, allowing for a more direct and objective evaluation of a child’s behavioral patterns and functional abilities. This approach is particularly advantageous in a virtual setting, providing a more accurate representation of everyday environments than traditional methods (14, 15).

Efforts have been made over the past several years to use VR technology to address the challenges noted above in diagnosing ADHD. The most widely used and researched areas is the application of VR to the Continuous Performance Test (CPT) (15). VR-CPT is an assessment tool that evolves the widely used neuropsychological test for ADHD evaluation, the CPT, using VR technology. Since 2007, when Parsons et el. (2007) study on VR-CPT were first introduced, implementing the CPT in a virtual classroom setting, this new concept of assessment tool has been gradually improved and continuously researched (15, 16). Initially, it involved using a head-mounted device to present visual and auditory stimuli and measure responses. However, research conducted by Iriarte et al. (2016) and Areces et al. (2018) introduced a classroom-based VR-CPT that enables movement tracking (17, 18). A meta-analysis published in 2019 reported that classroom-based VR-CPT reliably distinguishes between the attentional performance of ADHD and control groups (19). VR-CPT enhances ecological validity compared to traditional CPT by consistently controlling distractions and conducting assessments in an environment similar to daily life. It also offers additional neuropsychological indicators through movement tracking that are not available in traditional CPT. However, the CPT task has limitations in assessing symptoms that occur in various activities in daily life.

To overcome the limitations of VR-CPT, Ryu et al. (2021) and Son et al. (2021) made initial attempts to use VR and AI to measure everyday activities in virtual reality for diagnosing ADHD (20, 21). They presented tasks that children at the elementary school level typically perform, such as packing a backpack, tidying a room, scheduling, and solving puzzles. Participants were allowed to freely explore the VR space and engage in activities beyond the tasks, which significantly increased the freedom of movement, allowing for the observation of responses in a more natural setting. Compared to previous studies, this approach enabled the collection of more comprehensive daily activity data through voice recording to verify verbal responses, as well as through eye tracking and dynamic movement measurements. Additionally, the biometric data collected were analyzed using an AI algorithm based on the DSM-5 diagnostic criteria and quantified in a manner corresponding to the conventional clinical ADHD Rating Scale, proving to be useful for clinical application. In 2022, Seesjärvi et al. also tried to measure ADHD symptoms and executive functions by analyzing goal-directed behavior in everyday living within a virtual environment with the “EPELI” (Executive Performance in Everyday Living) device (22). They quantified behavioral data from performing various household chores in a virtual living space, presenting a new device that measures attention, executive function, and prospective memory in virtual reality.

This study is based on the research by Ryu et al., 2021 and Son et al., 2021 (20, 21). We innovatively utilized Virtual Reality (VR) to replicate everyday scenarios that children commonly experienced. This approach involved developing a novel cognitive assessment software, initially named “VR Quest” and later renamed to “AttnKare-D”, which leveraged behavioral data within VR environments to assist in the diagnosis of ADHD. The VR system comprised a Head-Mounted Display (HMD) and two controllers for interactive engagement, enabling children to perform tasks mimicking real-life activities. The HMD offered immersive visual and auditory stimuli, isolating the user from external distractions. Children interacted with the VR environment, performed tasks analogous to everyday situations, and transitioned to subsequent scenarios either upon task completion or as time progressed. This methodology highlights the potential of VR in enhancing diagnostic accuracy by simulating real-world contexts in a controlled setting.

Behavioral data within the VR environment were captured utilizing a tri-component system: a Head-Mounted Display (HMD) and a pair of controllers. Initially, the system recorded the three-dimensional (x, y, z) coordinates of each device at a frequency of 0.1 seconds. This enabled a precise quantification of the child’s physical movements and evaluation of associated behavioral manifestations. In the second phase, the interactive nature of controllers was employed, allowing children to execute both relevant and irrelevant actions within the VR environment. This functionality was instrumental in assessing their attention to assigned tasks and response latency and in identifying potential hyperactive tendencies through their spontaneous or prolonged physical engagements. Lastly, continuous voice recording of the child during task execution provided a crucial dimension for analyzing speech-related symptoms, thereby enriching overall behavioral evaluation in the context of ADHD.

To convert collected behavioral data into diagnostic information, each behavior observable in VR was categorized into 18 items per symptoms listed in DSM-5 ADHD Criterion A. These items were evaluated following the framework of K-ARS-IV (Korean version of ADHD Rating Scale, 4th edition), which utilized the DSM’s 18 symptoms. K-ARS-IV, which adapts these criteria, is a questionnaire that enables observers to assess symptom severity on a scale from 0 to 3. Accordingly, AttnKare-D gives severity scores ranging from 0 to 54 points. The list of behaviors anticipated to be observable in VR was developed with reference to DSM-5, ARS, DIVA-5, and CBCL. It was further refined through interviews with elementary school teachers. After finalizing the list under the advisement of three child and adolescent psychiatrists, we matched VR-observable behaviors with the 18 symptoms. Based on the VR behavior-ADHD symptom matching list, digital measurements obtained through VR equipment were matched with the behavior list, enabling conversion into ARS scores. Observed behaviors were scored based on their frequency and intensity. This process involved identifying key elements from a large data set, for which we implemented an AI model using a Convolution Neural Network (CNN) structure known for feature extraction. In our previous research, using an AI model, we constructed and analyzed a virtual dataset based on measurements from five researchers performing VR tasks, achieving 98.3% accuracy in distinguishing ADHD (21). Subsequently, the AI model was refined using data from over 2000 children who experienced AttnKare-D and their parents’ K-ARS survey responses.

In this study, we conducted an exploratory clinical trial to test the efficacy and safety of AttnKare-D, a novel ADHD diagnostic aid combining VR and AI, in children with and without ADHD. Our objectives were to: 1) establish AttnKare-D’s ADHD diagnostic cut-off value by comparing it to the gold standard (diagnosis by child and adolescent psychiatry specialists), 2) evaluate AttnKare-D’s diagnostic performance by examining the Area Under the Curve (AUC) value derived from its Receiver Operating Characteristic (ROC) curve against the gold standard, and 3) monitor any adverse reactions that might occur during AttnKare-D assessment.

Our research domain construct centers on the innovative integration of Virtual VR and AI to advance the diagnostic process for ADHD. By embedding VR technology into the diagnostic framework, we create simulated environments that mimic real-life situations, which are instrumental in capturing the naturalistic behaviors of children potentially affected by ADHD. This VR setup allows for an immersive and controlled observation of behaviors that conventional diagnostic methods might miss. Alongside VR, we employ AI algorithms to analyze the collected behavioral data with high precision. The AI component categorizes and quantifies these behaviors according to DSM-5 criteria, enhancing the objectivity of the diagnostic process. Together, these technologies form AttnKare-D, a diagnostic tool designed to not only overcome the subjective limitations of traditional ADHD assessments but also improve the accuracy and reliability of diagnoses. This comprehensive approach aims to set a new standard in ADHD diagnostics, providing a replicable model for future clinical applications and research in psychiatric evaluation tools.

## 2 Materials and methods

### 2.1 Participants

This study was conducted from March 2023 to June 2023 at two institutions, Samsung Medical Center and Samsung Changwon Hospital, where elementary students aged 6 to 12 years were recruited. Children with ADHD were enrolled based on core symptoms such as inattention, hyperactivity, and impulsivity during outpatient visits. The control group was recruited through hospital announcements. Exclusion criteria were children currently on or recently treated with ADHD medications (Methylphenidate, Atomoxetine, Clonidine, Guanfacine) within the past month and those who started or were undergoing cognitive-behavioral therapy for ADHD symptom relief in the last three months. Additionally, children diagnosed with major psychiatric disorders, developmental disorders, obsessive-compulsive disorder, severe depression or anxiety disorders, conduct disorder, or post-traumatic stress disorder were excluded. Those with an overall Intelligence Quotient (IQ) below 70 as measured by the Korean Wechsler Intelligence Scale for Children(K-WISC) were also excluded.

### 2.2 Ethical statements

This clinical trial was conducted after obtaining approval from the Korea Food & Drug Administration (Approval No. 1444) in accordance with Article 10 of the Medical Device Act and Paragraph 4 of Article 20 of its enforcement rule. Trial details were registered with the National Institute of Health’s Clinical Research Information Service (KCT0008403). Ethical approvals were obtained from the Institutional Review Boards of Samsung Medical Center (2022-03-051) and Samsung Changwon Hospital (2023-02-008). Researchers obtained written consent from participants and guardians after thoroughly explaining this study’s purpose and potential events during the study, adhering to the Declaration of Helsinki (established in 1975, amended in 2013).

### 2.3 ADHD diagnosis and clinical assessment

Child and adolescent psychiatrists conducted clinical interviews with child participants and one of their primary caregiving parents using the K-SADS-PL (Korean version of the Kiddie Schedule for Affective Disorders and Schizophrenia for School-Age Children - Present and Lifetime). After these interviews, psychiatrists evaluated the severity of symptoms using K-ARS. The primary caregiver was also administered the K-ARS and K-CBCL to measure the child’s ADHD symptoms with internalizing and externalizing problems. The child participant underwent the K-WISC and the Conners CPT-3 (Conners Continuous Performance Test 3rd edition). These assessments were used to determine cognitive levels and measure attention consistency, stability, and impulsivity in a controlled environment. Based on interview and psychological test outcomes, psychiatrists differentiated between children with ADHD and those without according to the DSM-5 diagnostic criteria.

### 2.4 VR assessment

For the VR component of this study, we utilized the Oculus Quest 2 manufactured by Oculus. This VR device comprises a single HMD and two hand controllers for individual operation. Visual stimuli in VR were presented using two display panels, one for each eye. Each panel had a resolution of 1832 x 1920 pixels. The child participant’s head movements and the corresponding changes in the VR environment had a minimal delay (in the milliseconds), typically imperceptible in terms of motion delay.

Child participants in the study wore the Head-Mounted Display (HMD) and held two controllers to perform various tasks in the VR environment, following the software’s guidance. These tasks included organizing a room, moving balls, spinning pedals, matching numbers, packing a backpack, planning schedules, and wrapping gifts. Adequate time for practice was provided before tasks to familiarize children with the VR equipment. The VR setting replicated common indoor environments such as school or home. Tasks were designed to reflect activities often done in school or home settings. Total duration for AttnKare-D varied among individuals. Each VR scenario had a time limit, allowing completion within 20-25 minutes. The activity took place on a flat 3-meter x 3-meter indoor area, where children moved freely while standing and performed tasks. The VR environment was confined to a space smaller than 3-meter x 3-meter. Although visually restricted from moving beyond, a researcher stayed close by for safety throughout the session. This careful design ensured a controlled yet realistic setting, providing a safe and effective environment for children to perform VR-based tasks.

**Figure 1:**
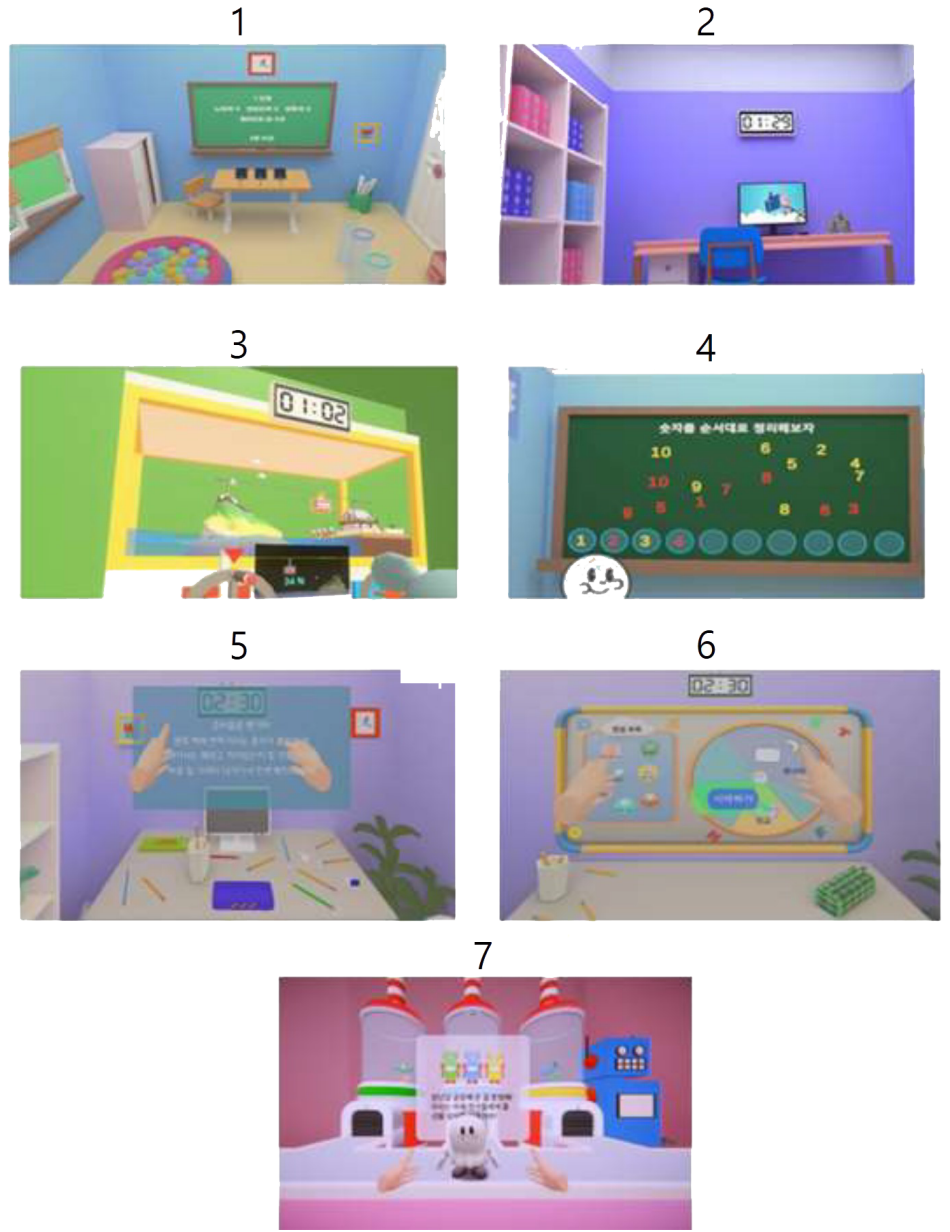
Cognitive and behavioral tasks in VR scenarios. 1, moving balls; 2, organizing a room; 3, spinning pedals; 4, matching numbers; 5, packing a backpack; 6, planning schedules; 7, wrapping gifts.

When participants first entered the virtual space, they met a cute ghost character who introduced them to the virtual environment and the tasks that would be performed, including how to walk within the space and operate the controller. If participants had trouble with the device operation, the examiner provided direct assistance. Only those who completed the tutorial tasks smoothly proceeded to the main tasks. Participants performed 4 out of 7 possible tasks, which were conducted in a random order considering the difficulty of each task and the age of the participants. In the Organizing Room task, participants were required to tidy up a cluttered room filled with numerous objects and trash within a set time limit, placing specific items in designated locations. During this task, distractions such as a video playing on a monitor and toy ducks moving around the floor were present. In the Moving Ball task, participants had to use a shovel to move balls into tubes matching the colors of the balls within a limited time. This task was presented in three levels of difficulty, with varying numbers of balls and spatial constraints. In the Spinning Pedals task, participants had to pedal at speeds matching the character’s speed to move a toy cable car to its destination. There were four levels of pedal speed, requiring participants to adjust their pedaling speed accordingly. In the Matching Numbers task, participants needed to find and sequentially arrange numbers from 1 to 10 on a chalkboard, alternating with the ghost character. This task was offered in two levels of difficulty, like the Children’s color trails test (CCTT), with participants needing to pay attention to the colors assigned to odd and even numbers. In the Packing a Backpack task, participants first packed school supplies into a pencil case and then packed the pencil case along with other items into a backpack. Between stages one and two, a mini task involved shooting a basketball into a hoop to assess participants’ ability to switch attention. The basketball shooting task was performed once before moving to the second stage, but failure to progress was scored. In the Planning Schedule task, participants arranged their desired plans on a circular schedule board in stage one, remembered and sequentially arranged these plans in stage two, and in stage three, they had to logically sequence tasks as if borrowing books from a library. In the Wrapping Gifts task, participants had to wrap a robot according to rules suggested by the ghost character. Among the robots, some were subtly defective and had to be sorted into a special box. This task was divided into three stages, each with different sorting rules and increasing difficulty (**Figure 1**). In all scenarios, performance efficiency for each task was measured based on accuracy and time taken. Additional behavioral data collected included the number of times and duration participants looked at unnecessary places or stayed in irrelevant locations, the frequency and duration of their gaze being diverted by distractive objects, and how often they touched distractive objects. Actions such as hitting other characters within the scenario were scored as disruptive behaviors. Throughout the duration of the tasks after the tutorial, voice recording was conducted, measuring the frequency of speech, the volume of the voice, and responses started before voice questions were completed, which were then scored according to related diagnostic criteria.

Behavioral data collected from each VR scenarios were calculated into z-scores. Their averages were then matched with the behavioral categorization list. Matched data percentages were computed and compared against ARS-IV norm percentages (5). This was followed by a distribution process, through which individual scores for each of the 18 symptoms and a total score were derived and formatted in the K-ARS form.

### 2.5 Statistics

**Figure 2:**
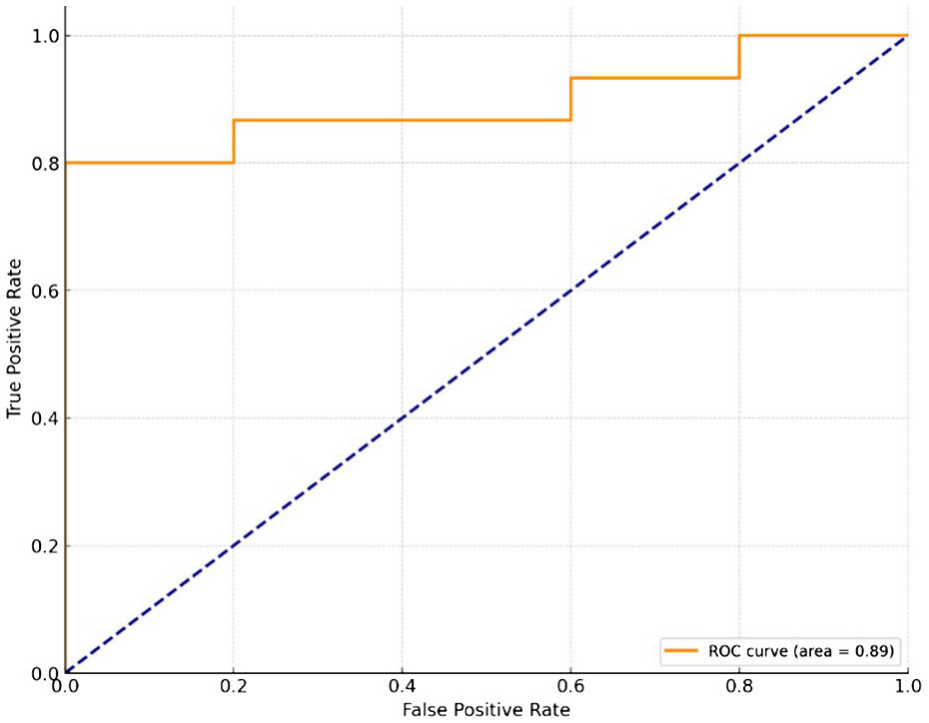
Receiver operating characteristic (ROC) curve. Sensitivity = True Positive Rate, Specificity = 1-False Positive Rate.

**Figure 3:**
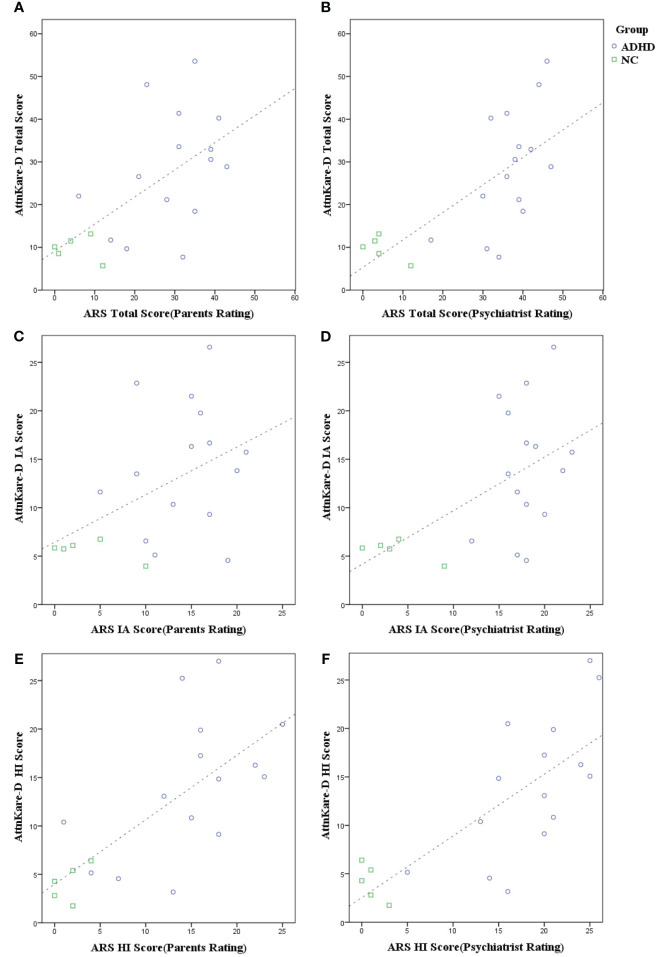
Correlation between ADHD symptom severity and VR task performance. Each graph compares K-ARS (Korean ADHD Rating Scale) scores by parents and psychiatrists with AttnKare-D scores, for total, inattentive, and hyperactive/impulsive categories. **(A)** Parents vs. AttnKare-D total scores, **(B)** Psychiatrists vs. AttnKare-D total scores, **(C)** Parents vs. AttnKare-D inattentive scores, **(D)** Psychiatrists vs. AttnKare-D inattentive scores, **(E)** Parents vs. AttnKare-D hyperactive/impulsive scores, **(F)** Psychiatrists vs. AttnKare-D hyperactive/impulsive scores.

The average of demographic data, clinical data, and the symptoms scores of ADHD were compared between the ADHD group and the Normal control group. Age, Full Scale Intelligence Quotient (FSIQ) measured by WISC, K-ARS score, K-CBCL score, and the result of CPT-3 were not assumed to be normally distributed for each group, so a nonparametric method, the Mann-Whitney test, was used to compare the means (**Table 1**). To validate the diagnostic performance of the device, a Receiver Operating Characteristic (ROC) analysis was conducted using the ADHD diagnosis by a child and adolescent psychiatrist and the scores derived from the device as variables (**Table 2**, **Figure 2**). Pearson correlation analysis was conducted to analyze the correlation between the rating scores obtained through the K-ARS questionnaire and the scores from AttenKare-D (**Table 3**, **Figure 3**). Results were considered statistically significant at p < 0.05. All statistical analyses were conducted using SPSS 24.0 software (IBM Corporation, Armonk, NY, USA).

**Table 1:** Demographics and clinical characteristics of children with ADHD (N = 15) and normal controls (N = 5).

Items	ADHD (M ± SD)	NC (M ± SD)	p
Age of years	8.13 ± 1.64	9.20 ± 1.30	.168
Male	12 (80%)	4 (80%)	
K-WISC-IV or V			
FSIQ	103.73 ± 14.66	103.40 ± 11.89	.933
K-ARS			
Total score	29.07 ± 10.70	5.20 ± 5.17	**.001**
Inattention	14.27 ± 4.62	3.16 ± 4.04	**.002**
Hyperactivity/impulsivity	14.80 ± 6.74	1.60 ± 1.67	**.001**
K-CBCL			
Total problems	67.27 ± 10.72	49.40 ± 7.77	**.001**
Internalizing problems	60.20 ± 10.44	52.20 ± 9.99	.197
Externalizing problems	65.00 ± 12.98	46.00 ± 6.67	**.002**
Conners CPT-3			
d’	55.13 ± 7.21	52.60 ± 6.66	.694
Omissions	57.73 ± 16.57	55.80 ± 9.83	.793
Commisions	50.93 ± 8.17	49.00 ± 6.67	.600
Perseverations	58.27 ± 14.02	52.00 ± 6.96	.511
HRT	56.27 ± 9.46	51.40 ± 12.76	.483
HRT SD	58.47 ± 13.00	52.40 ± 6.62	.512
Variability	55.59 ± 10.87	54.00 ± 9.92	.541
HRT Block Change	50.38 ± 12.14	47.80 ± 12.91	.662
HRT ISI Change	52.73 ± 9.00	51.60 ± 9.40	.861

NC, Normal Control; M, Mean; SD, Standard Deviation; N, Number; K-WICS-VI or V, Wechsler Intelligence Scale for Children 4th edition or 5^th^ edition; FSIQ, Full Scale Intelligence Quotient; K-ARS, Korean version of the ADHD Rating Scale; K-CBCL, Korean version of the Child Behavior Check List. The bold text indicates p < 0.05.

**Table 2:** Receiver operating characteristic (ROC) analysis and data points of the ROC curve.

Cut-off value	Sensitivity	Specificity
54.57	0.000	1.0
53.57	0.067	1.0
18.44	0.800	1.0
13.15	0.800	0.8
11.71	0.867	0.8

**Table 3:** Correlation coefficients between parents, psychiatrists, and AttnKare-D rating scores with significance levels.

ARS items	Parents_ Psychiatrists	Parents_AK	Psychiatrists_ AK
Total	0.86^**^	0.62^**^	0.70^**^
IA	0.85^**^	0.45	0.56^*^
HI	0.85^**^	0.72^**^	0.77^**^
1	0.62^**^	0.31	0.34
2	0.63^**^	0.53^*^	0.67^**^
3	0.61^**^	0.37	0.39
4	0.65^**^	0.62^**^	0.78^**^
5	0.69^**^	0.45	0.62^**^
6	0.65^**^	0.69^**^	0.69^**^
7	0.48^**^	0.44	0.47
8	0.65^**^	0.63^**^	0.62^**^
9	0.40	0.46^*^	0.39
10	0.52^*^	0.45	0.65^**^
11	0.49^*^	-0.02	0.18
12	0.69^**^	0.74^**^	0.68^**^
13	0.39	0.14	0.46^*^
14	0.51^*^	0.56^*^	0.77^**^
15	0.67^**^	0.73^**^	0.62^**^
16	0.82^**^	0.72^**^	0.71^**^
17	0.30	-0.12	0.49^*^
18	0.87^**^	0.61^**^	0.60^**^

Correlation coefficients were calculated using Pearson’s method. Asterisks denote significance levels: * for p ≤ 0.05 and ** for p ≤ 0.01.

In the ARS Questionnaire, odd-numbered items (e.g., 1, 3, 5, etc.) pertain to inattentiveness, while even-numbered items (e.g., 2, 4, 6, etc.) relate to hyperactivity/impulsivity.

Parent_Psychiatrist, Correlation between parent and psychiatrist responses; Parent_AK, Correlation between parent responses and AttnKare-D AI analysis; Psychiatrist_AK, Correlation between psychiatrist responses and AttnKare-D AI analysis; IA, Inattentive sub-total score; HI, Hyperactive/Impulsive sub-total score. The bold text indicates p < 0.05.

## 3 Results

### 3.1 Participants and clinical characteristics

A total of 21 children were enrolled in this study. Of these, 16 were diagnosed with ADHD and 5 were healthy controls. One child diagnosed with ADHD who withdrew consent during the study was excluded from the analysis, resulting in 15 children in the ADHD group and 5 in the control group participating in this study. There were no dropouts due to adverse reactions.

Age and overall intelligence showed no significant differences between the two groups. In both groups, 80% were males, reflecting the gender distribution typical for ADHD diagnoses in this age range (23). The average K-ARS total score for the ADHD group was 29.07 ± 10.70, surpassing the diagnostic cutoff of 19. The two groups showed statistically significant differences in K-ARS total scores (*p* = 0.001), inattention (*p* = 0.002), and hyperactivity/impulsivity (*p* = 0.001). For clinical symptoms assessed by K-CBCL, the ADHD group had higher scores of total behavior problems (67.27 ± 10.72) and externalization (65.00 ± 12.98), both above their clinical ranges, while their internalization scores (60.20 ± 10.44) were in the subclinical range. The control group scored within normal ranges for all CBCL parameters. The ADHD group had significantly higher scores for total problem behaviors (*p* < 0.001) and externalized behaviors (*p* < 0.001), but not for internalized symptoms (*p* = 0.197). However, Conners CPT measurements showed no significant differences between the two groups (**Table 1**).

### 3.2 Diagnostic performance of AttnKare-D

AttnKare-D can be used to score ADHD symptoms in the same 18-item format as K-ARS. ROC analysis was conducted to validate AttnKare-D’s diagnostic performance by comparing it with the final diagnosis made by child and adolescent psychiatrists. AttnKare-D showed optimal diagnostic performance with a sensitivity of 0.8 and a specificity of 1.0 at the cutoff score of 18.44, closely matching with K-ARS’s diagnostic cutoff of 19 (24) (**Table 2**). The area under the ROC curve (AUC) indicates diagnostic performance, with a higher value signifying a better diagnostic accuracy. The AUC scale can be interpreted as follows: AUC = 0.9–1.0, excellent; AUC = 0.8–0.9, very good; AUC = 0.7–0.8, good; AUC = 0.6–0.7, sufficient; AUC = 0.5–0.6, bad; AUC < 0.5, test is not useful (25). AttnKare-D’s AUC value in the ROC analysis was 0.893, classifying it as very good (**Figure 2**).

### 3.3 Correlation between K-ARS and AttnKare-D

This study analyzed correlations between K-ARS scores rated by parents or child and adolescent psychiatrists and scores derived by AI based on data collected in the VR environment. AttnKare-D showed a statistically significant positive correlation with K-ARS score assessed by parents (r = 0.62, *p* < 0.01) or psychiatrists (r = 0.70, *p* < 0.01). However, different patterns emerged in symptom-specific scores. For hyperactivity/impulsivity scores, AttnKare-D had a strong positive correlation with K-ARS score rated by parents (*r* = 0.72, *p* < 0.01) or psychiatrists (r = 0.77, *p* < 0.01). For inattention scores, AttnKare-D showed a moderate positive correlation with psychiatrist-rated K-ARS score (r = 0.56, *p* < 0.05) and a trend towards correlation with parents-rated K-ARS score (r = 0.45, *p* = 0.051), although such trend was not statistically significant (**Table 3**, **Figure 3**). The K-ARS is structured such that odd-numbered items can assess inattention and even-numbered items can assess hyperactivity/impulsivity. In item-by-item analysis comparing AttnKare-D with K-ARS scores, parents-rated scores showed no significant correlation for eight specific items (seven odd-numbered and one even-numbered), while psychiatrists-rated scores showed no significant correlation for five specific items (all odd-numbered) (**Table 3**).

## 4 Discussion

AttnKare-D, a novel digital medical device, can predict ADHD diagnosis by analyzing behavioral data digitalized in a VR environment simulating real-life settings. Its AI-based analysis showed a cutoff score (18.44 points) close to the reference standard of K-ARS (19 points) for parents’ assessments of children’ clinical symptoms, demonstrating a commendable diagnostic performance (AUC = 0.893). To the best of the authors’ knowledge, studies that integrally use VR and AI to predict ADHD diagnosis through real-life behavioral patterns have not been reported yet.

Among various diagnostic aids for measuring ADHD symptoms, the Continuous Performance Test (CPT) is widely used. Recent meta-analysis studies have reported that the AUC of CPT for diagnosing ADHD across 19 studies ranges from 0.7 to 0.8 (26), which is lower than that found in the present study.

Results of the Conners CPT for the children in our study did not predict ADHD diagnosis, an unexpected outcome that was different from previous research. This discrepancy might be attributed to the relatively smaller sample size of participants in our study than in previous research, potentially impacting the statistical significance.

AttenKare-D measures ADHD symptoms based on everyday activities, thus offering the advantage of assessing children’s everyday behavior in a naturalistic and ecologically valid manner compared to VR-CPT studies. While both use head-mounted devices and auditory stimulation to provide a virtual environment, previous VR-CPT studies measured attention based on more static movements and task performance (15). In contrast, AttenKare-D allows for greater freedom of movement within the set virtual space, which is beneficial for measuring more dynamic movements. The vast amount of movement data accumulated during scenarios, whether related to the task or not, has been advantageous for hyperactivity analysis using AI. Recent advancements in eye-tracking tasks using VR-CPT and the analysis through machine learning are showing a trend towards the enhancement of neuropsychological information that can be provided in a controlled environment (27). As will be discussed later, inattention in this study was found to have a lower correlation with caregiver and clinician ratings compared to hyperactivity/impulsivity symptoms, suggesting that future eye-tracking analysis in VR-CPT could be a valuable technique to complement the performance of AttenKare-D.

In terms of collecting and analyzing data from everyday activities within virtual reality, the EPELI study can be considered similar to our own VR assessment tool (22, 28). Participants use an HMD and hand controllers to navigate and perform household tasks within a virtual apartment, where EPELI is able to quantify and present the measured behaviors. In a study comparing 38 ADHD patients with typically developing controls, ADHD group exhibited lower attentional-executive efficacy and more controller and game action movements, distinguishing the two groups with an AUC of 0.88, a diagnostic performance similar to our study’s ability to differentiate the groups (28). Both EPELI and AttnKare-D meticulously measured participants’ movements; however, AttnKare-D categorized movement data according to DSM-5 diagnostic criteria and adjusted weights using an AI algorithm to present results in a format similar to the traditional ARS questionnaires, while EPELI set detailed metrics such as navigation efficacy, number of correctly performed tasks, overall actions, time monitoring, and controller movement. The differences in analytical methods and result presentation could impact the convenience and extensibility in clinical applications. AttnKare-D offers convenient results based on the DSM-5, familiar to clinicians, and its AI model’s performance in analyzing participant behavior can improve rapidly as more data is collected, although its use is limited beyond ADHD diagnosis. On the other hand, while EPELI may lack convenience in clinical application for ADHD diagnosis, it can present neuropsychological results for executive function and memory function, suggesting a relatively broader scope of application for the future, beneficial for expansion into treatment and education. Furthermore, AttnKare-D has incorporated a voice recording and analyzing system to measure talkativeness and blurting out answers, which appears to be a clear strength compared to previous studies.

In this study, while AttnKare-D’s scores showed strong correlations with K-ARS scores for total and hyperactivity/impulsivity symptoms, these correlations were insignificant or weak for inattention symptoms. A detailed item-by-item analysis revealed that many items related to inattention lacked significant correlation (**Table 3**). Firstly, this could be due to difficulty in evaluating DSM-5-defined inattention symptoms using digitally measured data, which requires integrated analysis including task performance in daily life settings. The VR setup could effectively capture data for hyperactivity/impulsivity symptoms, as it could easily measure and analyze space movement and speech. Hyperactivity-related items (items 2, 4, 6, 8, 10, and 12) in K-ARS, such as item 2 describing inability to stay still and constant movement, were aptly captured and analyzed using spatial movement data of VR equipment. Item 10 describing continuous movement as if driven by a motor aligned well with VR’s tracking capabilities. Additionally, item 12 about excessive talking could be quantitatively assessed through HMD’s voice recording feature. Impulsivity-related items 14, 16, and 18 involving behaviors such as answering questions prematurely or inability to wait for one’s turn were also amenable to analysis through VR by utilizing performance data in specific contexts. However, inattention symptoms, often less observable and requiring inference from task performance, presented challenges in scoring. Items in K-ARS such as item 1 “often fails to give close attention to details” and item 3 “has difficulty sustaining attention in tasks” which had low correlations with AttnKare-D scores highlighted this challenge. These items required an integrated analysis of task performance levels, response times, unnecessary actions, responses to instructions, task abandonment frequency, and gaze handling. Such behaviors and responses might not directly align with DSM-5’s described attention deficits. In contrast, item 15 “is often easily distracted by extraneous stimuli” showed significant correlations possibly due to its easier observation in specific VR scenarios. This variance suggests that while VR can be effective in measuring certain ADHD symptoms, particularly those observables in specific settings, it may not capture all aspects of inattention as defined in the DSM-5. Therefore, AttnKare-D’s scoring system might have limitations in fully representing the spectrum of inattention symptoms in ADHD. Secondly, attitudes of participating children towards VR equipment and environments might have influenced objective measurements. Most children readily accepted and even showed enthusiasm for VR testing. Given their age and tendency of children, especially those with ADHD symptoms, to be curious about new device, this engagement might have affected the accuracy of measuring attention levels. Children’s strong immersion in VR tasks, often more engaging than everyday activities, might have led to an underestimation of their usual attention levels. Specifically, for item 11 of the K-ARS about avoiding mentally demanding tasks, there was almost no correlation with AttnKare-D scores (r = 0.02 for parents, r = 0.18 for psychiatrists), possibly due to children’s over-involvement in the VR environment. This suggests a need for future task adjustments considering boredom and difficulty levels to better assess attention deficits. Thirdly, while VR-based assessments could simulate everyday life, they still carry limitations of a cross-sectional evaluation. Children with ADHD often exhibit fluctuating attention levels influenced by environmental factors, physical condition, and types of tasks. Hence, testing conducted on a specific day for a duration of 15-20 minutes might not fully capture a child’s overall attention span. On the other hand, symptoms of hyperactivity and impulsivity tend to be less variable. This aspect of the study highlights the need for a more comprehensive approach to evaluate inattention in VR environment.

In this study, the AttnKare-D developed initially under the guidance of a child and adolescent psychiatry specialist was designed to assist in conventional diagnoses. Thus, evaluations and analyses were conducted focusing on behavioral items observed in traditional diagnostic tools such as K-ARS, DIVA, and CBCL known to be based on DSM-5 symptom description. Essentially, during the development process, a psychiatrist advised on various specific behaviors typically observed in daily life of children with ADHD. These behaviors were then digitalized, categorized, and scored in the format of K-ARS. This could be seen as a form of supervised learning, where the psychiatrist provided a list of anticipated behaviors in children with ADHD for the AI model to learn from. This approach is suitable for the purpose of AttnKare-D in classifying ADHD diagnoses, as it allows for a level of classification accuracy, as revealed in this study. It uses human guidance for the development of AI, thereby providing diagnostic assistance in a format familiar to clinician. However, during the research, we observed several behaviors or symptoms that were not anticipated by the psychiatrist but proved helpful in predicting ADHD diagnosis. For instance, many ADHD children exhibited aggressive behaviors such as hitting characters that were designed to present tasks in a familiar manner. Some children attempted to catch objects (like small yellow ducks wandering on the floor in VR) designed to distract attention, moving around the room. VR scenarios were entirely set indoors. However, in some scenarios, it was possible to look outside through windows. Several children were observed to only stare at outdoor VR landscapes or persistently attempt to go outside, failing to complete their tasks. This continued even after the examiner mentioned that exiting outdoors was not possible in the VR setting. These observations suggest the need to further train the AI to include such behaviors in the analysis, highlighting a limitation of the expert-guided supervised learning approach. Conversely, adopting an unsupervised learning approach to investigate patterns and structures of children’s behaviors in VR, particularly those with ADHD, without predefined criteria could uncover abnormal behaviors related ADHD diagnosis that traditional diagnostic standards might overlook. This type of analysis, unlike conventional questionnaires or laboratory testing, could provide richer information about the behavior of children with ADHD to clinicians, a topic that must be considered in the future use of VR in the psychiatric field, particularly for behavioral issues.

Using VR that simulates real-life scenarios for the assessment and diagnosis of ADHD symptoms presents several clear advantages. Firstly, it allows for more objective measurement by eliminating subjective elements of observers. In traditional clinical settings, understanding the child’s real-life behavioral characteristics often relies on information filtered through perspectives of adults such as parents and teachers. When direct measurement of behavior in a VR environment is converted into explicit numerical values for assessment, it can minimize subjectivity. Secondly, it enables observation of behaviors in a setting that resembles real life rather than a clinical environment. Most children diagnosed with ADHD are in lower grades of elementary school, typically aged between 7 and 10 years. The unfamiliar clinic environment can either inhibit or, conversely, disinhibit a child’s behavior symptoms. Most child and adolescent psychiatric settings attempt to establish a comfortable environment using toys and drawing materials to alleviate these issues, yet there are inherent limitations. In this study, children showed high levels of engagement in the VR environment. Even those who were somewhat reserved in the clinic exhibited full immersion during VR assessments. Thirdly, observing children’s behaviors in VR is advantageous for identifying various problematic behaviors that may manifest in real life but are not evident in clinical settings or included in diagnostic criteria. As previously mentioned, AttnKare-D applies traditional diagnostic standards in a scoring format similar to ARS, making it challenging to capture various problematic behaviors not initially included in primary measurements. However, following future development directions, it is expected to provide additional behavioral information. This will be crucial for delivering personalized medicine, a recent trend in healthcare. Behavioral patterns can vary widely even among children with the same ADHD diagnosis. Identifying behaviors unique to each individual that hinder daily functioning can greatly aid clinicians in determining the most appropriate treatment approach.

No adverse reactions that are commonly anticipated with the use of VR devices, such as headaches, dizziness, eye strain, eye or muscle twitching, involuntary eye movements, nausea, discomfort or pain in the head or eyes, sleepiness, and fatigue, were reported in this study. Additionally, simulator sickness, often occurring in previous VR studies due to head movement and time delays in scenes projected by head-mounted devices, was not observed in this study. Such issues have become increasingly rare in recent studies due to the improved speed of modern VR devices compared to earlier generations (29). AttnKare-D appears to be relatively stable for application in children. However, this study involved only 20 children, which is a relatively small sample size; therefore, safety should be further observed in future studies involving larger groups.

This study has several limitations. Firstly, the total number of participants was 20, with only 5 in the control group. This small sample size could lead to overrepresentation of individual behavioral characteristics in results and limit statistical analysis. Furthermore, dividing the participants into ADHD children and a control group was influenced by the small sample size. Additionally, the fact that the number of children with ADHD and the control group were not equal, with a ratio of 3:1, must be considered when interpreting the results. However, this study was an Exploratory Clinical Trial preceding a Pivotal Clinical Trial for medical device approval by the Ministry of Food and Drug Safety of South Korea, aimed at assessing clinical utility and diagnostic cutoff of the device with a small number of participants. Secondly, this research was conducted at two institutions in Seoul and Changwon involving only Korean participants and Korean language users, making it difficult to generalize findings to other ethnicities and language groups. Thirdly, we were unable to adjust for differences in participants’ proficiency with VR devices. Although most children were using VR for the first time, there was a considerable variation in their adaptability to the device. The impact of device familiarity on task performance could not be completely ruled out. However, to control for this, ample explanation and practice time were provided before task an, with examiners beginning assessment only when they deemed children sufficiently familiar with the VR device. Fourthly, both fourth and fifth editions of the Wechsler Intelligence Scale for Children (K-WISC) were used. Due to changes in assessment items and scoring methods in the fifth edition, comparative analysis of specific subtests other than overall intelligence was not feasible.

In conclusion, we tested the efficacy and safety of a novel digital diagnostic device that uses VR technology to observe behavior and provides information about ADHD symptoms in a form that can be utilized in existing medical practice through an AI analysis model. This study, the first clinical application of AttnKare-D, involved 20 children and showed that AttnKare-D has a diagnostic performance with an AUC of 0.893 at a cut-off value of 18.44. No discomfort or adverse reactions were observed during the VR assessment process. To confirm the diagnostic utility of AttnKare-D, it is necessary to conduct clinical trials with a sufficient number of participants and to validate its use across different ethnic and cultural backgrounds internationally. Additionally, improvements in the accuracy of inattention symptom evaluation within the analysis model are required.

## Data availability statement

The raw data supporting the conclusions of this article will be made available by the authors, without undue reservation.

## Ethics statement

The studies involving humans were approved by Institutional Review Boards of Samsung Medical Center (2022-03-051) and Institutional Review Boards of Samsung Changwon Hospital (2023-02-008). The studies were conducted in accordance with the local legislation and institutional requirements. Written informed consent for participation in this study was provided by the participants’ legal guardians/next of kin.

## Author contributions

SO: Conceptualization, Data curation, Formal Analysis, Investigation, Writing – original draft. YJ: Conceptualization, Data curation, Investigation, Project administration, Supervision, Validation, Writing – review & editing. TC: Conceptualization, Funding acquisition, Project administration, Resources, Software, Writing – review & editing. JL: Data curation, Investigation, Writing – review & editing. BS: Data curation, Investigation, Writing – review & editing. NK: Data curation, Software, Writing – review & editing. HS: Data curation, Software, Writing – review & editing.
